# mTOR-Dependent Spine Dynamics in Autism

**DOI:** 10.3389/fnmol.2022.877609

**Published:** 2022-06-15

**Authors:** Shabani Chaudry, Nandini Vasudevan

**Affiliations:** School of Biological Sciences, University of Reading, Reading, United Kingdom

**Keywords:** autism spectrum conditions, social behaviors, rodent models, spine density, synaptic transmission, neurocircuitry, mTORC1, autophagy

## Abstract

Autism Spectrum Conditions (ASC) are a group of neurodevelopmental disorders characterized by deficits in social communication and interaction as well as repetitive behaviors and restricted range of interests. ASC are complex genetic disorders with moderate to high heritability, and associated with atypical patterns of neural connectivity. Many of the genes implicated in ASC are involved in dendritic spine pruning and spine development, both of which can be mediated by the mammalian target of rapamycin (mTOR) signaling pathway. Consistent with this idea, human postmortem studies have shown increased spine density in ASC compared to controls suggesting that the balance between autophagy and spinogenesis is altered in ASC. However, murine models of ASC have shown inconsistent results for spine morphology, which may underlie functional connectivity. This review seeks to establish the relevance of changes in dendritic spines in ASC using data gathered from rodent models. Using a literature survey, we identify 20 genes that are linked to dendritic spine pruning or development in rodents that are also strongly implicated in ASC in humans. Furthermore, we show that all 20 genes are linked to the mTOR pathway and propose that the mTOR pathway regulating spine dynamics is a potential mechanism underlying the ASC signaling pathway in ASC. We show here that the direction of change in spine density was mostly correlated to the upstream positive or negative regulation of the mTOR pathway and most rodent models of mutant mTOR regulators show increases in immature spines, based on morphological analyses. We further explore the idea that these mutations in these genes result in aberrant social behavior in rodent models that is due to these altered spine dynamics. This review should therefore pave the way for further research on the specific genes outlined, their effect on spine morphology or density with an emphasis on understanding the functional role of these changes in ASC.

## Introduction

Autism Spectrum Conditions (ASC) or Autism Spectrum Disorders (ASD) are a group of neurodevelopmental disorders defined by the DSM-VI by the presence of two core symptoms: persistent deficits in social communication, social interaction, socio-emotional reciprocity, and restricted repetitive patterns of behavior, interests, or activities (Baron-Cohen, 2009; Mandy et al., 2012). Commonly diagnosed at ages 2–3, ASC affects around 1% of the population, with ASC traits recently understood to be continuous within the general population albeit those diagnosed with ASC being at the extreme end of this spectrum (Lenroot and Yeung, 2013; Ruzich et al., 2015). The elevation of these continuously distributed ASC traits in first degree relatives of those with ASC suggests a genetic basis for ASC (Piven et al., 1997; Eyuboglu et al., 2018). This genetic basis for ASC is useful to identify critical molecular and cellular drivers that may underlie the behavioral deficits in ASC. This review seeks to: (a) identify mTOR (mammalian target of rapamycin) and specifically the mTORC1 complex as a central convergent pathway for a group of select high risk ASC genes; (b) summarize how these genes can regulate a cellular phenotype, i.e., spine dynamics *via* mTORC1 mediated pathways; and (c) how spine dynamics may in turn result in the behavioral and synaptic physiology phenotypes that are seen in ASC. We do so by delineating supporting correlative evidence in human subjects in Section “Functional Connectivity in ASC Is Linked to Spine Dynamics” and “Many Genes Linked to ASC Are Regulators of mTORC1 or Targets of mTORC1” and use data from rodent models of ASC in further sections in order to explore genetic causality for both spine dynamics and behavior.

## Functional Connectivity in ASC Is Linked to Spine Dynamics

### Functional Connectivity Within the DMN Underlies the ASC Phenotype

ASC is understood to be a whole brain disorder, whose neural correlates range from alterations in synapses and neural transmission to brain volume and structure. Alterations in functional connectivity have long been implicated in the pathophysiology of ASC since differences in functional connectivity have been found in an estimated 90% of studies (Lau et al., 2013; Chen et al., 2015; Mohammad-Rezazadeh et al., 2016) although the direction of this difference, implicated brain areas, brain state, and age are disputed (Müller et al., 2011; King et al., 2019). A dominant but challenged theory (Picci et al., 2016) is the “Cortical Underconnectivity Theory” which states that there is long range underconnectivity particularly between hemispheres and frontal-posterior brain areas in ASC individuals which may explain symptomatology (Just et al., 2012). In support of this theory is the finding that many ASC individuals have decreased Corpus Callosum (CC) volume (Frazier and Hardan, 2009), and that both adults and children with congenital agenesis of the CC (AgCC) show deficits in social and emotional intelligence and score highly on the Autism Quotient (Lombardo et al., 2012; Lau et al., 2013) without intellectual disability (Lábadi and Beke, 2017). Furthermore, fMRI studies of individuals with AgCC show decreased long range functional connectivity, suggesting that decreased long range connectivity between hemispheres is what contributes to ASC symptomology (Owen et al., 2013).

Apart from underconnectivity driven by the corpus collosum, underconnectivity could also be seen by activity in the nodes of the Default Mode Network (DMN), a set of nuclei that are preferentially activated, as visualized by rsfMRI (resting state magnetic resonance imaging) scanning, while individuals are not focused on the external environment (i.e., “mind wandering” or “resting state”). While DMN activation ceases once cognitive effortful tasks commence (Anderson et al., 2011; Li et al., 2014; Padmanabhan et al., 2017), mind wandering involves internally generated thoughts about others, one’s self and remembering past or envisioning future events, as elucidated by designed self-report questionnaires and interviews (Andrews-Hanna et al., 2013; Smallwood and Schooler, 2015). The DMN identified in this manner consists of the medial prefrontal cortex (mPFC), the posterior cingulate cortex, precuneus, medial temporal lobe (which includes the hippocampus), and inferior parietal cortices (Li et al., 2014; Alves et al., 2019) which together incorporate memories (hippocampus) and emotions (mPFC) in order to create “mind wandering” (Poerio et al., 2017). Hence, adults with bilateral ventromedial prefrontal cortex (vmPFC) lesions show a reduction in self-reported mind wandering (Bertossi and Ciaramelli, 2016).

Decreased connectivity is often detected by fMRI in ASC individuals within the DMN, with underconnectivity having been found positively correlated to ASC social deficits, increased repetitive stereotyped behaviors measured by Autism Diagnostic Observational Score (ADOS; Yerys et al., 2015) as well the Autism Diagnostic Interview-Revised (ADI-R; Chen et al., 2021b). Though this data is necessarily correlative in nature, long range underconnectivity in DMN areas could be a neuroanatomical signature for ASC individuals who could then be tested for social deficits (Nair et al., 2020) using the Theory of Mind (ToM) tasks in which ASC individuals show marked deficits (Frith and Happé, 1994). However, other studies have shown short range, long range and overall brain connectivity to be functionally overconnected in adults (Belmonte, 2004; Kleinhans et al., 2016), though long range connections to and from the frontal cortex appear to be consistently under-connected (Courchesne and Pierce, 2005), with strength of connectivity between the PFC and right parietal cortex negatively correlated with autism symptom severity (Redcay et al., 2013). However, long range hyperconnectivity between the thalamus and auditory and somatosensory cortices as well as parietal regions has also been found in fMRI studies of adults with ASC (Tomasi and Volkow, 2019).

Varying long range connectivity in studies could occur due to the polygenetic nature of ASC with different genetic rodent models showing different functional connectivity patterns. Therefore, it may be possible to use varying patterns of connectivity as an endophenotype for identifying different forms of ASC (Zerbi et al., 2021). For example, homozygous knockout male mouse models of Fragile X Syndrome (FXS) show long range cortico-striatal connections to be functionally underconnected (Zerbi et al., 2018), whereas heterozygous knockout male TSC models mouse models show the same connections to be functionally hyperconnected (Pagani et al., 2021). This long range hyperconnectivity found within the TSC mouse model was also found to be associated with increased repetitive behaviors in the mice. Increased repetitive behaviors and increased long range hyperconnectivity are both reversed by the administration of the mTOR inhibitor rapamycin, which functionally reverses the effects of the loss of one of the two *Tsc2* genes (Pagani et al., 2021).

Conversely, individuals with depression present increased rumination and self-referential mind wandering (Nejad et al., 2013; Nayda and Takarangi, 2021) that is correlated with hyperconnectivity between the long distance DMN nodes mPFC and the posterior cingulate cortex, suggesting that the DMN nuclei must maintain optimal connectivity for healthy cognition and perception of self (Wise et al., 2017). In humans the use of ketamine, a compound that is primarily an NMDA receptor antagonist (Sleigh et al., 2014) has been shown to alleviate symptoms of depression, as well as reduce functional connectivity within the DMN of healthy individuals (Scheidegger et al., 2012) and those with Major Depressive Disorder (MDD; Evans et al., 2018), suggesting that many behaviors are strongly correlated with functional connectivity. These studies show that functional connectivity in the DMN may be a marker of behaviors that denote increased or decreased self-reference.

### Synaptic Physiology Underlies the Differences in Connectivity in ASC Individuals

What underlies differences in DMN connectivity in ASC individuals? Within discrete brain areas, this may be attributed to an Excitation Inhibition (E/I) imbalance and reduction in signal-to-noise ratio in neural circuitry that is implicated in ASC (Rubenstein and Merzenich, 2003; Testa-Silva et al., 2012). Indeed, the high incidence of epilepsy within the ASC population (Viscidi et al., 2013) as well a postmortem study showing upregulation of excitatory AMPA receptors (Purcell et al., 2001), may indicate that there is an increased excitation within the brain of ASC individuals. How increased excitation within the brain of ASC individuals causes local or long distance hyperconnectivity or hypoconnectivity is not fully understood though changing E/I ratios in local circuits to understand this question is now underway in a number of rodent models of ASC (See Section “Molecular Mechanisms That Link Dysfunctions in Neuromorphology to Behavior”). Interestingly, hyperconnectivity between various modes of the DMN, including that detected in a Tsc2^+/−^ mouse model of ASC using rsFMRI is linked to an excess of mTOR signaling and increased spine density in the insular cortex. Using *in silico* modeling, these increases in synapse density were linked to hyperconnectivity and use of a mTORC1 inhibitor, rapamycin, reversed both the increases in spine density and the hyperconnectivity in this model (Pagani et al., 2021). This strongly suggests that connectivity in the DMN is positively correlated to spine density which is in turn driven by mTORC1.

### Spine Dynamics Contributes to the E/I Ratio Within Neural Circuits

Clearly, DMN connectivity and the underlying E/I ratio could be dependent on neuronal architecture including dendritic spine dynamics reflected by spine density and spine morphology (Gao and Penzes, 2015). Dendritic spines are typically around 0.5–2 microns long, dendritic protrusions that receive signals from axonal boutons of other neurons to form synapses allowing neurons to transmit signals to one another (Horner and Arbuthnott, 1991). Human postmortem studies reveal that synapse formation increases throughout childhood from birth in both ASC and Typically Developing (TD) children, but is slightly elevated in ASC (Penzes et al., 2011). Synapses are then pruned from adolescence into adulthood, with pruning being more efficient in TD over ASC subjects, leaving ASC individuals with a greater number of synapses. In adulthood, this is followed by an equilibrium in synapse formation and elimination to maintain a stable number of synapses in TD individuals (Rakic et al., 1986; Masliah et al., 1993; Peters et al., 1998; Penzes et al., 2011); there is increased synapse maintenance during adulthood in ASC, often leading to a greater number of spines and synapses compared to TD individuals (Penzes et al., 2011). The typical development of cortical dendritic spines in rodents follows a similar pattern to humans, with an initial increase in dendritic spine density during the first two postnatal weeks, followed by an increase in spine pruning over spine formation during adolescence, with spine density then stabilizing in adulthood (Bhatt et al., 2009). Apart from spine number, the shape of spines is also important (Table 1). New immature spines have a small surface area and postsynaptic density (PSD), with decreased number of receptors; this could reflect an increased “neuroplasticity” of neural circuits, with a greater potential for strengthening, thus making them “learning” spines (Bourne and Harris, 2007). It should be noted that the classification of filipodia spines as being a defined and individual spine type or just the precursor to dendritic spines is disputed (Takahashi et al., 2003; Kanjhan et al., 2016); this is due to the dynamic nature of filipodia-type spines which give rise to all other spine types. However, since many studies investigating dendritic spine dynamics in rodent models of ASC report filipodia-type spines as part of the immature dendritic spine classification, and report changes in the quantity of filopodia type spines in various rodent ASC models, we have also decided to report filipodia type spines as a distinct spine classification within the larger category of immature spines (Dictenberg et al., 2008; Williams et al., 2008; Dunaevsky et al., 2014; Pyronneau et al., 2017; Sceniak et al., 2019; Skelton et al., 2020). Wider more mature “memory” spines have greater surface area and PSD for receptors and form very strong synapses with very little range for the synaptic strengthening/adaptation that is associated with Long Term Potentiation (LTP), on increased stimulation (Cooke and Bliss, 2006). Spine shape and consequently maturity are characterized by their length and width measurement ratios (Harris and Kater, 1994), with immature spines, i.e., filopodia, thin, long thin spines tending to be longer and thinner in size and mature spines, i.e., mushroom and stubby shorter and wider (Table 1). An increase in mushroom type mature spines and increase in overall spine width in the primate prefrontal cortex is associated with cognitive decline during aging in rhesus macaques (Dumitriu et al., 2010), suggesting inflexibility and loss of neural plasticity (Bourne and Harris, 2007, 2008).

**Table 1:** Characteristics of dendritic spines.

Spine type	Maturity	Shape	Prevalence	Function	References
Filipodia 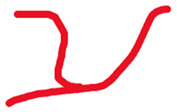	Immature, motile, learning spines existing for 4 days or less	Long, thin and no real head neck configuration	65% of total spines in human adult cortex and hippocampus and most prevalant spine type in rodent brains	Spines originate as filipodia in order to find synaptic partners on nearby dendrites and invoke spine formation. They then shorten and widen as they mature. Smaller surface area, so smaller postsynaptic density, Associated with Long Term Depression, synaptic plasticity and learning	Peters and Kaiserman-Abramof (1970), Harris and Kater (1994), Nimchinsky et al. (2002), Holtmaat et al. (2005), Cooke and Bliss (2006), Bourne and Harris (2007), Bourne and Harris (2008), Dumitriu et al. (2010), Berry and Nedivi (2017), Jawaid et al. (2018), and Parker et al., 2020)
Long Thin 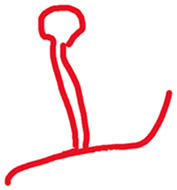		Long (not as long as filipodia) and thin with head neck configuration
Thin 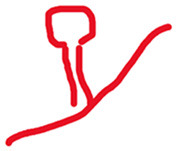		Long (shorter than both Filipodia and long thin) with head neck configuration
Stubby 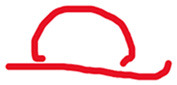	Mature, memory spines existing for 8 days or more	Wide (not as wide as mushroom) and short	25% human adult cortex and hippocampus and 2nd most prevalent type in rodent brains	Associated with Long Term Potentiation, decreased neuroplasticity, ageing
Mushroom 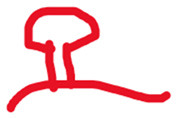		Widest spine type and short
Branched 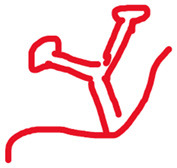		Two heads from one base	Around 10% of spines in human adult cortex and hippocampus and least prevalant spine type in rodent brains	Unknown	

*Dendritic spines on neurons can be of many shapes, with stubby and mushroom-shaped spines typically thought of as mature and thin, filamentous spines thought of as immature and more motile (Risher et al., 2014). Different spine shapes have been implicated in different functions*.

A recent study using living humans showed that synaptic density (which may be indicative of spine density), as measured by PET scanning of synaptic glycoprotein 2A, is negatively correlated with symptom severity in adults with MDD, within the dorsolateral prefrontal cortex (Holmes et al., 2019). Such local hypoconnectivity is inversely related while long range hyperconnectivity between the dorsolateral prefrontal cortex and the posterior cingulate cortex is positively correlated with depression symptoms of clinically depressed individuals, suggesting that the underlying measurements at the synapse, i.e., spine density/morphology may have predictive value. In support of this idea, multiple rat models of depression also show a decrease in dendritic arbor within the hippocampal CA3 (Watanabe et al., 1992) and mPFC pyramidal cells (Goldwater et al., 2009), both key nodes involved in the DMN. Furthermore, ketamine inhibited dendritic spine loss in chronically stressed rats that displayed symptoms of depression *via* protection of mushroom shaped spines, and through the induction of spine formation (Ng et al., 2018).

Human postmortem studies, using Golgi-Cox staining, have shown an increase in spine density in ASC individuals compared to controls, independent of sex, within the amygdala, cortex, and temporal lobes (Hutsler and Zhang, 2010; Tang et al., 2014; Weir et al., 2018; Table 2). This increased spine density in ASC individuals (Weir et al., 2018) is correlated with increased head circumference/macrocephaly, as well as enlargement of specific brain areas including the amygdala and hippocampus (Schumann et al., 2004; Tilot et al., 2015; Weir et al., 2018). Only one human postmortem study assessed spine morphology *via* Golgi-cox staining in ASC vs. TD controls and found that ASC individuals had more “compact” spines with decreased spine length within the cortex, which indicates a more mature spine morphology (Hutsler and Zhang, 2010; Table 2). This along with the increased spine density found in ASC individuals may reflect an increase in local connectivity linking spine density to functional connectivity. One thing to note is that increased head size (macrocephaly) is a common finding in children and adults with ASC; toddlers with ASC show accelerated brain growth compared to TD controls to possibly result in an enlarged amygdala and hippocampus during adolescence (Groen et al., 2010) with the degree of amygdala enlargement being a predictor for ASC symptom severity (Schumann et al., 2004). A mouse ASC genetic model of Phosphatase and tensin homolog (*Pten*) haploinsufficiency also reflects the macrocephaly found within ASC individuals (Huang et al., 2016). Furthermore this model shows increased brain enlargement and macrocephaly that is correlated with hyperconnectivity and dendritic arborization (Huang et al., 2016), suggesting that ASC individuals with macrocephaly may also have similar hyperconnectivity with increased spine densities. A conditional knockout of *Pten* in a subset of auditory cortical neurons shows enhanced local and global hyperconnectivity with the strength of synaptic inputs from the thalamus and auditory neurons increased; this was decreased by the mTOR inhibitor rapamycin (Xiong et al., 2012). Together, these studies suggest that functional connectivity is correlated with both spine density and mTOR signaling and both these neural correlates may result in macrocephaly.

**Table 2:** Brains from human ASC individuals show increased spine density.

Reference	Model	Sex	Age (years)	Brain area	Spine density	Spine morphology
Weir et al. (2018)	Postmortem human ASD	Males (*n* = 10) Females (*n* = 10)	7–46	Amygdala	Increased	Not tested
Tang et al. (2014)	Postmortem human ASD	Males (*n* = 18) Females (*n* = 2)	3–19	Temporal lobe	Increased	Not tested
Hutsler and Zhang (2010)	Postmortem human ASD	Males (*n* = 10)	10–45	Cortex	Increased	Increased mature
						type spines

*Postmortem brains were obtained and stained by Golgi staining, with spines counted in the amygdala, cortex, and temporal lobe (Hutsler and Zhang, 2010; Tang et al., 2014; Weir et al., 2018). Only one study investigated spine morphology and showed an increased proportion of mature mushroom-shaped spines (Hutsler and Zhang, 2010)*.

## Many Genes Linked to ASC Are Regulators of mTORC1 Or Targets of mTORC1

Though twin and genome studies have demonstrated that ASC has a genetic component, the genetic basis could be syndromic or non-syndromic. Though syndromic forms account only for 2%–4% of ASC cases, they implicate genes that can then be used to understand underlying cellular processes (Sztainberg and Zoghbi, 2016; Ziats et al., 2021) in manipulatable rodent models. These genes tend to be very rare within the general population but they have a large effect size in that they are very penetrant (Geschwind, 2011). Therefore, syndromic forms of ASC are highly comorbid with heritable, monogenic autosomal dominant disorders caused by the dysfunction of one identified gene or chromosome section with high penetrance; examples include Fragile X syndrome (FXS), Tuberous Sclerosis Complex (TSC), Phelan-McDermid syndrome (PMS), Rett Syndrome, Smith Lemli Opitz syndrome, and Angelman/Prader-Willis Syndrome (Fernandez and Scherer, 2017). For example, 61% of TSC patients have ASC (Vignoli et al., 2015). Non-syndromic forms of ASC could be due to the mutation of many common genetic variants or Single Nucleotide Polymorphisms (SNP’s) which along with epigenetic factors summate in order for ASC symptoms to reach a threshold for diagnosis (Rylaarsdam and Guemez-Gamboa, 2019; Satterstrom et al., 2020). These genes being prevalent in the general population means that they are present in many healthy individuals and may contribute to normal variation of traits, that they have a small effect size and individually do not often result in the development of ASC in the individual. However, it is the combination of different SNP’s along with various epigenetic factors that may lead to ASC diagnosis (Geschwind, 2011). Despite several genes implicated in ASC, 75%–95% of ASC cases are idiopathic having an unidentified genetic basis (Caglayan, 2010; Richards et al., 2015; Garg and Green, 2018). The Simons Foundation Autism Research Initiative (SFARI) database contains a repository of ASC-associated genes which are categorized as either “Syndromic” (S), 1, 2 or 3 according to the strength of evidence (stronger → weaker) linking the gene to ASC (for more information see https://gene.sfari.org/about-gene-scoring/; Banerjee-Basu and Packer, 2010).

**Figure 1:**
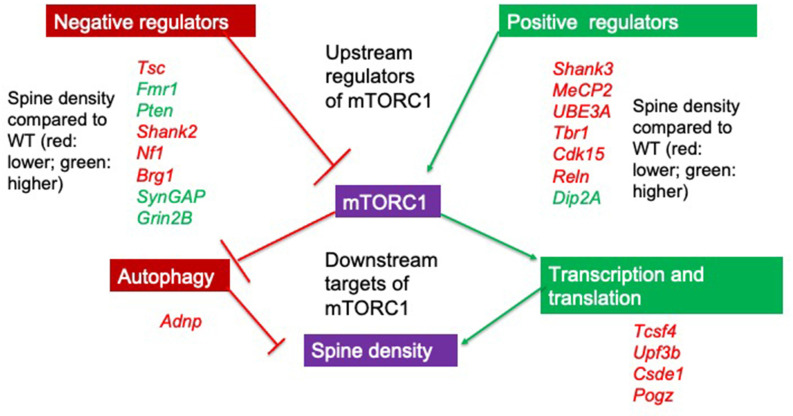
Upstream regulators and downstream targets of mTORC1. Positive and negative regulators of mTORC1 signaling show alterations in spine density when deleted in murine models. All identified genes with a SFARI score of 1 can regulate mTORC1 and all change spine density compared to wildtype (WT) animals. If spine density is increased compared to WT, this is denoted by the gene in green while if spine density is decreased compared to the WT, this is denoted by the gene in red. Note that positive regulators of mTORC1 (with the exception of *Dip2A*) all decrease spine density either when deleted or when duplicated (UBE3A and MeCP2). Though we do not focus on downstream targets of mTORC1 in this review, these targets are involved in transcription, translation, and autophagy. *Adnp*, Activity dependent neuroprotective protein; *Tcsf4*, Transcription factor 4; *Upf3b*, Regulator of nonsense transcripts 3B; *Csde1*, Cold shock domain-containing protein E1; *Pogz*, Pogo transposable element with ZNF domain.

Many ASC-linked genes have been implicated in synapse development through regulating spinogenesis (Sztainberg and Zoghbi, 2016; Satterstrom et al., 2020). Using integrated transcriptomics analyses of microarray and RNA-seq data from a number of rodent ASC models, proteins expressed at glutamatergic synapses are preferentially identified as altered in ASC (Duan et al., 2019). Both spine density and dendritic arborization (Copf, 2016) have been identified as molecular processes in neurons that are important for several neurodevelopmental disorders, including ASC (Nishiyama, 2019). Together, these studies suggest that dysregulation of spine density may be causal to autistic behaviors. Since in rodents key genes and neuromorphological alterations associated with ASC are conserved (Varghese et al., 2017) with humans, and behavioral tasks such as the social discrimination and social interaction tasks and the marble burying task that mimic the ASC phenotype are well established (Crawley, 1999, 2007), focusing on genes that affect spine density or spine morphology in rodents may lead to mechanistic insight (Lazaro and Golshani, 2015) of how these can affect behavior. In order to do so, we searched the available literature on PubMed to identify 141 genes identified using the search terms “spine density” or “spine morphology” AND “autism” or “ASC” in animal models. Out of 80 genes that showed differences from the wildtype (WT) genotype in mean spine density and/or morphology, 20 have a score of 1 in the SFARI database. All these 20 genes are either regulators of the mTORC1 complex or are regulated by the mTORC1 pathway (Figure 1), a major convergent pathway for syndromic and nonsyndromic genes (Magdalon et al., 2017). Though other pathways, notably those involved in cell adhesion may also be important, we concentrate solely on the upstream regulators of mTORC1 signaling with a focus on studies where spine dynamics is altered in this review. We also discuss their effects on neuromorphology and ASC-type behaviors in genetically altered rodent models and infer mTORC1 dependent and independent molecular processes driven by them that may cause the alterations in neuromorphology and behavior.

## The mTORC1 Pathway in ASC Is A Critical Hub for Both Neuromorphology and Behavior

**Figure 2:**
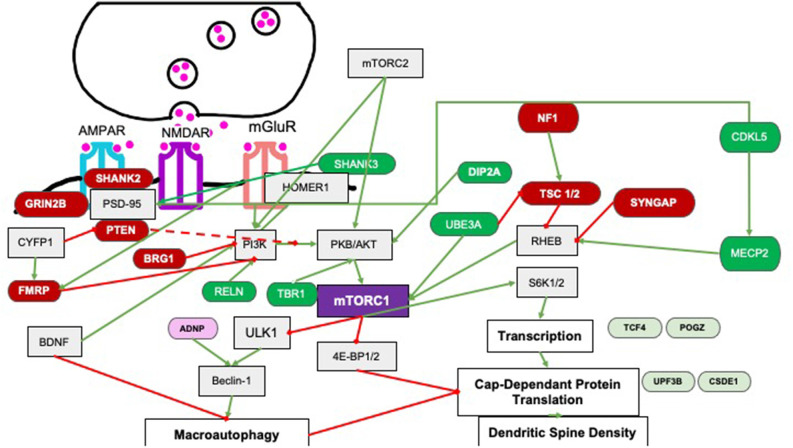
Pathways by which upstream regulators affect mTORC1 signaling. Several regulators affect mTORC1 signaling *via* PI3K signaling or *via* Rheb signaling. Green and red-labeled genes represent ASC related genes that are upstream regulators of mTORC1 in an either positive (dark green) or negative (dark red). Downstream target genes linked to ASC that show changes in spine density are shown as light green (positively regulated by mTORC1); or pink (genes negatively regulated by mTORC1). Downstream target genes of mTORC1 are not the focus of this review. Green arrows indicate activation and red lines represent inhibition.

mTORC1 is a serine/threonine kinase within the PI3K-related kinase (PIKK) family, made up of mTOR, Raptor, and mLST8 (mammalian lethal with Sec13 protein 8; Saxton and Sabatini, 2017) subunit and is sensitive to rapamycin inhibition. A major upstream regulator is PI3K-phosphorylated Protein Kinase B (PKB) which directly activates mTORC1 and indirectly does so by inactivating an inhibitor, i.e., the Tuberous Sclerosis 1 and 2 (TSC1/2) proteins which in turn normally inhibit Rheb GTPase from activating mTORC1 (Ersahin et al., 2015; Figure 2). In cells, the mTORC1 pathway is a critical nexus in cells linking external stimuli such as energy availability, stressors, and abundance of growth factors as well as activation of glutamate receptors (AMPA/NMDA) and associated proteins at the synapse (SYNGAP/HOMER/SHANK/PTEN) to downstream increases in protein translation and maintenance of autophagy (Winden et al., 2018). Downstream signaling targets include S6K which upregulates translation initiation and elongation *via* eIF4A and eEF2 respectively, as well as increases cell growth and cell survival (Ersahin et al., 2015). The mTORC1 pathway maintains autophagy by inhibiting ULK-1, an activator of BECLIN which is required for the formation of the autophagosome (Jhanwar-Uniyal et al., 2019); hence overactivation of mTORC1 results in a decrease in autophagy.

In several models of autism such as deletions or mutations of PTEN, TSC, NF1 or FMRP proteins, mTORC1 activity is increased (Magdalon et al., 2017 and references therein). For example, tuberous sclerosis complex genes 1 and 2 (*Tsc 1/2*) code for the protein hamartin and tuberin respectively which heterodimerize to inhibit RheB, which normally directly activates mTORC1 (Franz and Capal, 2017). Therefore deletion, mutation or rearrangement of the *Tsc* genes leads to a loss of activity of the complex and overactivation of the mTORC1 pathway (Huang and Manning, 2008; Portocarrero et al., 2018). As expected, proteomic analyses show increases of mTOR in both the hippocampus and striatum of *Tsc1*^+/−^ mice (Wesseling et al., 2017) and increases of downstream targets such as *Ulk1* mRNA and phospho-S6K protein in the *Tsc2*^+/−^ mouse brain (Sato et al., 2012). This shows that mTOR overactivation by the deletion of an ASC-associated gene can increase the expression of downstream targets of mTORC1 (Figure 2). Since mTORC1 overactivation in this manner causes increases in spine density that is correlated with DMN hyperconnectivity (Pagani et al., 2021), other upstream regulators of mTORC1 may also alter behavior *via* spine density alterations.

### Autophagy, Downstream of mTORC1 Signaling, Is Required for Optimal Spine Density and Social Behavior

If mTORC1 signaling is a critical nexus for upstream regulators that are deleted or mutated in autism and if spine dynamics are an underlying mechanism for autism, then it is plausible that loss or mutation of upstream regulators may affect spinogenesis and/or spine pruning *via* mTORC1 signaling, just as it does social behavior (Section “Rapamycin, an Inhibitor of mTORC1, Reverses Autistic-Like Behaviors”). In ASC individuals, postmortem studies show a decrease of LC-III, an autophagosome marker, suggesting decreased pruning. Decrease of this marker is correlated with increased spine density and persistent PSD-95 marker expression in the temporal lobe throughout childhood and adolescence (Tang et al., 2014). Supporting this, a neuronal deletion of *atg7*, a E1 ligase required for phagosome formation in mice results in social interaction deficits and spine density on basal dendrites that stays elevated on cortical pyramidal neurons into adulthood. A second murine model that shows the significance of spine pruning is the *Tsc2*^+/−^ haploinsufficient mouse where reduction of this negative regulator of mTORC1 leads to increases in spine density in cortical projection neurons (Tang et al., 2014). Though rapamycin rescues both behavioral deficits and aberrant spine density in the *Tsc2*^+/−^ mouse, it does not do so in the *Atg7* conditional knockout (cKO) mouse or in the double mutant *Tsc2*^+/−^, *Atg7*^cKO^ mouse, demonstrating an absolute need for spine pruning in order to ameliorate social behavior deficits (Tang et al., 2014). A second upstream negative regulator of mTORC1 is the Fragile X mental retardation protein (FMRP; Sharma et al., 2010; Hoeffer et al., 2012; Darnell and Klann, 2013; Casingal et al., 2020), the *Fmr1* KO shows increased spine density in the hippocampus which in turn is correlated with higher long term depression (LTD) and a lack of discrimination in the novel object recognition task. These phenotypes are ameliorated with shRNA to Raptor, an unique component of the mTORC1 complex, suggesting that overactivation of mTORC1 specifically leads to the cognitive deficit (Yan et al., 2018). This rectifying effect of decreasing Raptor levels could be abrogated by decreasing ATG7, a protein required for phagosome formation, suggesting that Raptor rescued spine and cognitive deficits *via* the activation of autophagy in hippocampal neurons (Yan et al., 2018). Consistent with the idea that rectifying mTORC1 activity is central to rescuing spine density and social behavior, ketamine, a mTORC1 activator because it also secondarily increases AMPA signaling, rescued the lack of fear memory and decreased spine density in the prefrontal cortex seen in a heterozygote mouse deficient in the RELN (reelin) protein. Since RELN is a positive activator of mTORC1, lack of this protein leads to lower activation of this pathway which can be rescued by activation with ketamine. Conversely, rapamycin, an inhibitor of mTORC1 can reverse the ketamine-mediated rescue of spine density and fear memory in the *Reln*^+/−^ mouse, suggesting that both neuromorphology and cognitive and social behaviors are highly sensitive to mTORC1 levels (Iafrati et al., 2013; Section “Rapamycin, an Inhibitor of mTORC1, Reverses Autistic-Like Behaviors” below).

### Rapamycin, an Inhibitor of mTORC1, Reverses Autistic-Like Behaviors

If mTORC1 is a critical nexus for ASC, we would also expect that alteration of this pathway results in behaviors that denote autism in rodent models. To give an example, haploinsufficiency of *Tsc2* or *Tsc1* in the cerebellum using a conditional knockout (KO) mouse model is sufficient for the manifestation of social preference in the 3-chamber preference test and repetitive behavior in the marble burying task and self-grooming with all behavioral phenotypes ameliorated with rapamycin treatment (Tsai et al., 2012; Reith et al., 2013). Similarly, a selective deficit in social interaction but not in locomotor or food exploration activity was noted in both *Tsc1* and *Tsc2* heterozygote mutant mice. This deficit is reversed in both genotypes by rapamycin (Sato et al., 2012). *Tsc2*^+/−^ mice also show decreased memory in the Morris Water Maze and no discrimination between novel and training contexts in the contextual fear conditioning task, behaviors that could possibly be due to unstable late-phase LTP at the Schaeffer collateral synapse in the hippocampus; these were also reversed by rapamycin. This is a specific effect since early phase LTP, basal synaptic transmission and paired pulse facilitation are unaffected (Ehninger et al., 2008). Rapamycin reversal of specific behavioral and synaptic phenotypes demonstrates that mTORC1 signaling is specifically critical in driving autism-like behaviors in rodents.

The consequence of overactivation of the mTORC1 pathway would be an increase in translation and hence we would expect to see similar behavioral deficits in mice that overexpress or show greater activity of the downstream targets of mTORC1. Mice that are deleted for the repressor e4B-BP1, mimic mTORC1 overactivation since they have increased eIF4E activity and show increased excitation-inhibition (E/I) ratio, suggesting altered synaptic properties in the CA1 of the hippocampus. They also show decreased vocalization and socialization and no preference for conspecifics in the 3-chamber test as well as increased anxiety and repetitive behaviors such as self-grooming (Gkogkas et al., 2012). Interestingly, pharmacological inhibition of eIF4E normalized both social and repetitive behaviors and the E/I ratio. Similarly, a transgenic mouse that overexpressed eIF4E mimicking ASC individuals with a SNP in the promoter of the eIF4E gene that increases the level of this protein, also showed increased repetitive behavior and anxiety, decreased contact with conspecifics and higher mEPSCs from pyramidal neurons in the prefrontal cortex. This could be due to an increased spine density through an increase of smaller spines (Santini et al., 2012). Again, intracerebroventricular infusion of the eIF4E inhibitor, 4EGI-1, improved social behaviors and decreased anxiety (Santini et al., 2012). In *Fmr1* (Fragile-X mental retardation 1) KO mice, where mTORC1 is overactive because of loss of inhibition of PI3K, S6K is also overactivated; deletion of S6K increased dendritic spine maturation with concomitant improvement in social interaction behaviors and mGluR-mediated long term depression (LTD; Bhattacharya et al., 2012). Taken together, all these studies suggest that overactivation of mTORC1 and S6K signaling is sufficient for the manifestation of autistic-type behaviors.

## Many Upstream Regulators of mTORC1 Are Expressed at The Dendrite Or at The Post-Synaptic Density (PSD)

Of the upstream regulators of mTORC1 identified (Tables s 3 and 4), 75% are expressed either at the membrane of neurons in the PSD or at the dendrite. These are RELN, SHANK3 (SH3 and ankyrin repeat domain protein), DIP2A, CDKL5, FMRP, SHANK2, PTEN, SYNGAP (Synaptic Ras GTPase-activating protein), GRIN2B (Glutamate ionotropic receptor NMDA type subunit 2B) proteins and include both positive and negative regulators of mTORC1 function. One percent of all ASC cases show mutations in the SHANK family of proteins which are scaffolding proteins at the PSD that link to PSD-95, Homer, Arc2/3, the WAVE regulatory complex, SHARPIN, and glutamate receptors (AMPA and NMDA) *via* intermediate molecules or directly *via* their several conserved domains (Naisbitt et al., 1999; Delling and Boeckers, 2021). The presence of most mTORC1 regulators at the synapse as well as the data in Section “The mTORC1 Pathway in ASC Is a Critical Hub for Both Neuromorphology and Behavior” using mutant mTOR regulator rodent models suggests that synaptic parameters such as spine density, spine morphology, dendritic arbor, and the functional correlate, i.e., synaptic physiology play an important and possibly causal role in autistic-like behaviors in the rodent models.

**Table 3:** Upstream regulators of mTORC1 implicated in ASC that act as positive regulators of mTORC1 signaling.

Gene	No. of articles	Average spine density	Spine morphology	Murine social beh.	Ultrasonic vocalizations	Repetitive beh.	Human syndrome	Microcephaly
SHANK3	9	D	Decreased mature and immature spine morphology	Decreased	Decreased	Inc. self grooming and marble burying	Phelan-mcDermid syndrome (PMS)	yes
MECP2	9	D	OE increased immature and decreased mature	Decreased	Altered	Inc. self grooming	Rett syndrome	yes
UBE3A	4	D	Increased immature decreased mature	Increased	Increased	Drecreased marble burying	Angelmans syndrome (AS)	yes
Tbr1	4	D	-	Decreased	Decreased	Increased self grooming	-	yes
CDKL5	1	D	Increased immature (filopodia, thin and stubby) decreased mature mushroom	Decreased	Decreased	Increased digging	CDKL5 defficiency syndrome	yes
RELN	1	D	-	Decreased	Decreased	-	-	yes
DIP2A	1	I	Increased mature (mushroom and stubby) and increased spine width and decreased length	Decreased	Decreased	Increased self grooming	-	-

*Loss of these genes typically (with the exception of *Dip2A*) results in decreased spine density and abnormal social behaviors and increased repetitive behaviors. Spine density is recorded as an mean of the spine density values reported in the hippocampus and/or cortex across several studies. Note that Angelmann’s syndrome in humans is a result of the deletion of the maternal allele of UBE3A while most ASC cases arise from a duplication of UBE3A. References: Fukuda et al. (2005), Dindot et al. (2008), Verpelli et al. (2011), Wang et al. (2011), Wang et al. (2014), Durand et al. (2012), Nguyen et al. (2012), Iafrati et al. (2013), Cochoy et al. (2015), Valluy et al. (2015), Kim et al. (2016), Mei et al. (2016), Okuda et al. (2017), Wang et al. (2017), Wang et al. (2019), Fuchs et al. (2018), Ma et al. (2019), Chen et al. (2020), Darbandi et al. (2020), and Jacot-Descombes et al. (2020)*.

**Table 4:** Upstream regulators of mTORC1 implicated in ASC that act as negative regulators of mTORC1 signaling.

Gene	No. of articles	Average spine density	Spine morphology	Murine social beh.	Ultrasonic vocalizations	Repetitive beh.	Human syndrome	Microcephaly
TSC	9	D	Increased immature	Decreased social recongnition memory	Decreased	Increased grooming and marble burying	Tuberous sclerosis complex	-
Fmr1	1	I	Increased immature and decreased mature	Decreased social recongnition memory	Decreased	Increased grooming and marble burying	Fragile X syndrome	yes
PTEN	5	I	Increased immature and mature	Decreased social recongnition memory	Drecreased	Increased grooming and marble burying	PTEN harmato ma tumour	yes
SHANK2	3	D	Increased immature and mature	Decreased social recongnition memory	Altered	Increased grooming	-	yes
Nf1	4	D	Increased immature and mature	-	-	-	Coffin-siris syndrome (CSS)	Microcephaly and macrocephaly
SYNGAP1	2	ND	Increased immature	Decreased preference for mouse over object	-	-	-	Microcephaly and macrocephaly
GRIN2B/Glun2B	2	I	Increased immature	No difference	No difference	Increased self grooming	grin2b-related neurodevelopmental disorder	Microcephaly and macrocephaly
CASPR2	2	D	Increased immature and mature	-	-	-	-	-

*Loss of these genes result in variable effects on spine density but all mouse models show abnormal social behaviors and increased repetitive behaviors. Spine density is recorded as an mean of the spine density values reported in the hippocampus and/or cortex across several studies. Note that there is only one study for Brg1 and hence this is not analyzed in the review. References: Tavazoie et al. (2005); Hayashi et al. (2007); Meikle et al. (2008); Berkel et al. (2010); Luikart et al. (2011); Wang et al. (2011); Anderson et al. (2012); Clement et al. (2012); Ginsberg et al. (2012); Henderson et al. (2012); Schmeisser et al. (2012); Bateup et al. (2013); Dolan et al. (2013); Haws et al. (2014); Oliveira and Yasuda (2014); Pop et al. (2014); Tang et al. (2014); Yasuda et al. (2014); Barnes et al. (2015); Nie et al. (2015); Spinelli et al. (2015); Sugiura et al. (2015); Williams et al. (2015); Varea et al. (2015); Hodges et al. (2017); Liu et al. (2017); Pyronneau et al. (2017); Cox et al. (2018); Jawaid et al. (2018); Yan et al. (2018); Arroyo et al. (2019); Booker et al. (2019); Gross et al. (2019); Sceniak et al. (2019); Skelton et al. (2019); Zhang et al. (2019); Banke and Barria (2020); Kulinich et al. (2020); Shih et al. (2020); Bland et al. (2021); Schaefer et al. (2021); and Sugiura et al. (2022)*.

### Positive and Negative Regulators of mTORC1 Have Opposing Effects on Spine Density and Synaptic Plasticity

Several reviews stress the role of mTOR signaling in autism (Magdalon et al., 2017; Winden et al., 2018) while others explore the role of spine dynamics in autism, focusing on a few genetic models (Copf, 2016; Lin et al., 2016; Martínez-Cerdeño, 2017). In these sections, we specifically focus on the effects of upstream positive and negative regulators of mTORC1 on spine density and/or spine morphology in rodent models and possible correlations with synaptic physiology [Section “Many Upstream Regulators of mTORC1 Are Expressed at the Dendrite or at the Post-Synaptic Density (PSD)”] and social behaviors (Section “Alterations of mTORC1 Activity by Alterations in the Expression of Upstream Regulators Result in Autistic Behaviors”).

#### Positive Regulators of mTORC1 at the PSD/Dendrite

Positive regulators of mTORC1 (Table 3) that are at the PSD/dendrite include SHANK3, DIP2A, RELN, and CDKL5. Two genes cyclin-dependent kinase-like 5 (CDKL5) encoded by the *Cdlk5* gene located on the X chromosome (Montini et al., 1998) and *Dip2A* (Disco-interacting protein 2 homolog A), located on Chromosome 21 respectively have an important role in neuronal development and migration (Chen et al., 2010) as well as synaptogenesis and spine maturation (Della Sala and Pizzorusso, 2014). CDKL5 is localized with RAC1 in the spine of postmitotic neurons (Chen et al., 2010) whereas DIP2A is at the cell membrane and PSD (Ouchi et al., 2010). CDKL5 stimulates mTORC1 signaling through positive regulation of MeCP2, another ASC-related gene (Tsujimura et al., 2015; Section “Effects of the Loss of ‘global’ Positive Regulators of mTORC1 on Spine Density and Synaptic Physiology”) while DIP2A is a binding partner of FSTL1, which mediates Akt/PKB phosphorylation (Ouchi et al., 2010; Liang et al., 2014) and therefore increases mTORC1 activity. Unusually, RELN is an extracellular ligand that binds to the ApoER2 and VLDLR to direct cortical neuronal migration (Jossin, 2020).

#### Effects of the Loss of Synaptic Positive Regulators of mTORC1 in the PSD on Spine Density and Synaptic Physiology

Heterozygote CDKL5 knockout mice show lower spine density on both apical and basal dendrites of pyramidal cells in the CA1 and dentate gyrus as well as decreased arborization as shown in lower number of branches and lower dendrite lengths (Trazzi et al., 2018). Despite a small increase in immature spines, this lower spine density is driven by a larger decrease in mature spines. Juvenile haploinsufficient reeler mice also show lower spine density, most of which is attributable to lower numbers of spines with smaller head widths, accompanied by a near absence of NMDA-dependent LTP (Iafrati et al., 2013). Unlike the other positive regulators, *Dip2A* KO mice show increased spine density but this is confined to basal dendrites with no change in spine density on apical dendrites in cortical pyramidal neurons. However, this increased spine density is due to an increase in stubby spines albeit and a decrease in mushroom spines, flattening the PSD and possibly decreasing mEPSC amplitude due to a smaller number of NMDA and GluR subunits at the PSD (Ma et al., 2019).

**Figure 3:**
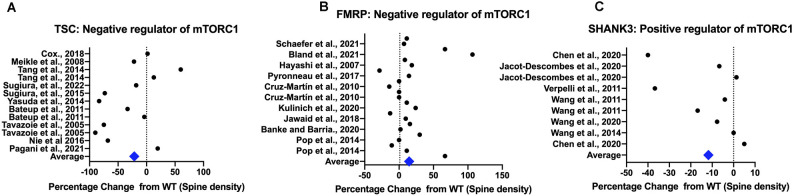
Rodent models show differences in spine density compared to controls. All spine density data from the TSC model are from *Tsc1/2* heterozygotes and KO animals (Panel **A**), for the FMRP model from *Fmr1* knockdown (KD) and knockout (KO) animals (Panel **B**) and for the SHANK3 model from *Shank3* knockdown (KD) and knockout (KO) animals (Panel **C**). TSC1/2 and FMRP are upstream negative regulators of mTORC1 while SHANK3 is a positive regulator. Spine density values from rodent studies was extracted using WebPlot Digitiser in a semi-quantitative fashion with the magnitude of the difference from the control plotted. The average difference for all models combined across cortex and hippocampus is shown, with all studies weighted equally. References: Tavazoie et al. (2005); Hayashi et al. (2007); Meikle et al. (2008); Verpelli et al. (2011); Wang et al. (2011); Wang et al. (2014); Durand et al. (2012); Ginsberg et al. (2012); Henderson et al. (2012); Bateup et al. (2013); Dolan et al. (2013); Pop et al. (2014); Tang et al. (2014); Yasuda et al. (2014); Cochoy et al. (2015); Nie et al. (2015); Sugiura et al. (2015); Sugiura et al. (2022); Mei et al. (2016); Hodges et al. (2017); Pyronneau et al. (2017); Cox et al. (2018); Jawaid et al. (2018); Yan et al. (2018); Arroyo et al. (2019); Booker et al. (2019); Gross et al. (2019); Banke and Barria (2020); Jacot-Descombes et al. (2020); Kulinich et al. (2020); Bland et al. (2021); and Schaefer et al. (2021).

Several homozygous and heterozygous rodent models use deletion or missense mutations of the scaffolding protein, SHANK, that mimic those found in human ASC individuals and that result in truncation of the protein. These are widely studied for their behavioral and neuromorphological phenotypes; for a detailed summary and a tabular synopsis of these, the reader is directed to Delling and Boeckers (2021) and Monteiro and Feng (2017) respectively. The best studied Shank isoform, SHANK 3 is uniquely expressed as part of the corticostriatal circuit in the striatum (Peça et al., 2011) and both a complete gene *Shank3* KO (Shank3^−/−^; Wang et al., 2016) and PDZ-domain deletions (Peça et al., 2011; Demir et al., 2016) show reduced spine density in the striatum. Mice with deletions of the ankyrin repeat domain of *Shank3* show reduced spine density in the hippocampus (Wang et al., 2011) and those with deletions in the Pro-rich (Zhou et al., 2016) domain also show reduced spine densities in the prefrontal cortex. In the hippocampus, LTP was also decreased in a *Shank3* mutant with a deletion of the ankyrin repeat domain (Jaramillo et al., 2016). Though some studies show no difference in spine density, the weight of evidence tends towards a decrease in spine density (Figure 3) with some studies reporting a decrease in mature mushroom shaped spines or spine width and length in a fairly domain-independent fashion (Wang et al., 2014). Local removal of *Shank3* in the nucleus accumbens reduces the firing rate in medium spiny neurons (MSN) of this region and whole *Shank3* KO displayed reduced sEPSC and LTD that is consistent with a reduced number of glutamatergic synapses on MSN in the striatum (Verpelli et al., 2011; Wang et al., 2016). However, the intrinsic striatal excitability was higher most likely due to increased glutamatergic drive from cortical afferents since hyperactivity is seen in the cortex of *Shank3*^−/−^ mice during a critical postnatal developmental period (PND14; Peixoto et al., 2016). These studies suggest that in general lower LTP or LTD along with lower spine density or less mature spines occur in mice that deleted for positive regulators of mTORC1.

#### Effects of the Loss of “Global” Positive Regulators of mTORC1 on Spine Density and Synaptic Physiology

Two other positive regulators of mTORC1 are MeCP2 (Methyl CpG binding protein 2), a transcriptional repressor and UBE3A (Ubiquitin-protein ligase E3A), a transcriptional coactivator and E3 ubiquitin ligase that targets proteins for degradation (Vatsa and Jana, 2018; Figure 2). In both cases, individuals presenting with increased dosage of these genes show symptoms of autism and hence overexpression and not deletion models of mice of these genes recapitulate features of autism. For example, the 2xTg-MeCP2 mouse which is a model for both Rett syndrome and ASC shows increased spine densities with a greater number of mushroom shaped spines, increased dendritic branching at younger ages that are normalized at around 40 weeks, possibly due to increased levels of S6K (Jiang et al., 2013). A PKA-phosphorylation site in UBE3A that is mutated in an ASC proband, T485A, also shows higher spine densities in cortical neurons transfected with the mutant, suggesting that overexpression of these positive regulators of mTORC1 increases spine density (Yi et al., 2015).

However, in some mouse models, overexpression of these genes leads to lower spine densities. For example, *MeCP2* overexpression in mouse cortical neuronal cultures interferes with the formation of the DiGeorge syndrome critical region 8 (DGCR8) complex with Drosha and lowers miRNA globally. This includes lowering the miRNA for CREB and LIM kinase (LIMK), whose levels rise; however, dendritic spine density decreases for reasons that are not clear (Cheng et al., 2014). Similarly, a study using inducible overexpression of *UBE3A* shows lower dendritic arbor, lower spine density with a lower proportion of mushroom shaped spines possibly due to an increased atypical pruning mechanism involving caspase (Khatri et al., 2018; Section “Upstream Regulators of mTORC1 Regulate Pruning by Several Pathways”).

Hence, though the loss of synaptically-localized positive regulators of mTORC1 typically leads to lower spine densities, smaller PSD and/or decreased sEPSCs/LTP, there is a lack of a clear pattern with more global positive regulators such as UBE3A and MeCP2 where overexpression rather than loss of the protein is present. This suggests that for global regulators, there are multiple targets which are critical rather than just overactivation of the mTORC1 pathway. This is exemplified by the MeCP2-mediated transcriptional repression of Reelin, where the decrease of Reelin in a MeCP2-overexpression ASC model may mimic the heterozygyous reeler mice and result in lower spine densities (Zhubi et al., 2014).

#### Negative Regulators of mTORC1 at the PSD/Dendrite and Their Effects on Spine Density and Physiology

Negative regulators include FMRP, SHANK2, PTEN, SYNGAP, GRIN2B, and TSC (Figure 2); many of the well-studied genes in this category when mutated or deleted in the mouse show increased spine density (Table 4) due to a combination of mTORC1-mediated lack of pruning and/or increase in spinogenesis pathways (Section “Upstream Regulators of mTORC1 Regulate Pruning by Several Pathways” and “Upstream Regulators of mTORC1 Regulate Spinogenesis by Several Pathways”).

The loss of PTEN, a negative regulator of the PI3K pathway, in hippocampal granule cells and cerebral cortex using a NSE-*cre* model leads to dendritic hypertrophy and macroencephaly in mice (Kwon et al., 2006). Consistent with this, a KO of *Pten* in granule cells at postnatal day (PND) 14 and PND 21 in a Brainbow mouse model results in longer apical dendrite lengths, greater distal arborization and increased soma size (Arafa et al., 2019). In support, E157G PTEN mutations found in ASC individuals when incorporated into iPSC-differentiated neurons also show an increase in dendritic arbor (Wong et al., 2020). This has functional implications—as expected, there is increased excitability in the postsynaptic dentate gyrus granule cell revealed by increased sEPSC, burst frequencies and reduced sIPSC in both male and females (Santos et al., 2017). This could also be due to excessive “wiring”—where competition by *Pten* KO cells is more efficient for the available pool of presynaptic partners resulting in increased glutamatergic excitatory afferents (Skelton et al., 2019). Similarly, truncation mutations of GRIN2B abrogated NMDA-dependent calcium influx and led to more filopodial spines in cortical neurons (Sceniak et al., 2019) even on a wildtype background, possibly due to a dominant negative effect. A third negative regulator is SynGAP which inhibits an activator of LTP, i.e., Ras-ERK signaling that in turns activates Rheb and mTOR, increasing translation of AMPA receptors (Groc et al., 2013). *SynGAP*^+/−^ mice show increased glutamatergic transmission when the perforant path is stimulated during an early postnatal period of hippocampal synaptic development (PND10-PND20) possibly due to an increased proportion of mushroom spines in dentate gyrus granule cells despite similar spine densities as wildtype mice. This accelerated development of glutamatergic synapses can also be seen with persistent propagation of signal in the hippocampal tri-synaptic circuit using photolysis of caged glutamate paired with fast voltage-sensitive dye imaging, demonstrating an increased E/I ratio (Clement et al., 2012). Similar stronger excitatory synapses and stronger basal transmission were seen in neurons derived from human iPSC cells where SYNGAP expression was reduced (Llamosas et al., 2020). A fourth model of a negative mTORC1 regulator is the mouse model of Fragile X syndrome (FXS) that also shows behaviors that mirror those seen in autism. Loss of the FMRP protein, that represses translation of other dendritic spine proteins, increases spine density in the medial prefrontal cortex, basal lateral amygdala, and hippocampus compared with wild type (Qin et al., 2011) with a greater proportion of thin spines and a lower proportion of mushroom spines (Jawaid et al., 2018) as well as hyperexcitability in the somatosensory cortex and exaggerated LTD in the hippocampus (Bhattacharya et al., 2012). This could be due to the reduction in a FMRP target calsyntenin-1 in the medial prefrontal cortex which in turn represses the synaptic protein ICAM5; an increase in ICAM5 in the *Fmr1* KO decreases spine maturation; overexpression of calysyntenin rescues spine maturation (Cheng et al., 2019).

**Figure 4:**
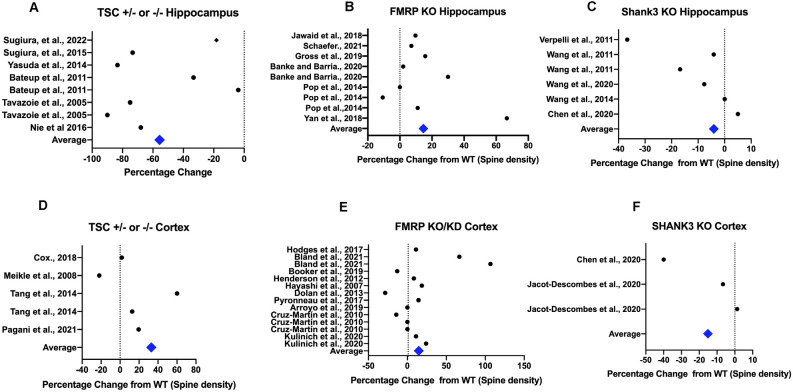
Spine dynamics depends on the brain area. Semi-quantitative plots show that spine density depends on the area studied, i.e., hippocampus **(A–C)** or cortex **(D–F)** for some commonly studied ASC-related genes that are either positive (*Shank3*) or negative regulators (*Fmr1, Tsc1/2*) of the mTORC1 pathway. All spine density data from these models were extracted using WebPlot Digitiser in a semi-quantitative fashion with the magnitude of the difference from the control plotted as percentage of control. The average difference from control WT animals for each model for each different brain area is shown, with all studies weighted equally (References in Figure 3).

Therefore overactivation of mTORC1 due to a loss of upstream negative regulators typically leads to an increase in spine density with a concomitant increase in excitability in local circuits. An outlier in this group is mice with *Shank2* PDZ domain mutations which show either a decrease in spine density (Won et al., 2012) or no effect (Schmeisser et al., 2012). This could be due to compensation by the increased expression of the SHANK3 isoform (Schmeisser et al., 2012) driving the spine density effect. Another notable exception is the TSC model where studies report variable effects (Figure 3) though ASC models of TSC show increased spine density in the cortex but not in the hippocampus (Figures 4A,D). This underscores that different brain regions may show different variations in spine density.

## Alterations of mTORC1 Activity by Alterations in The Expression of Upstream Regulators Result in Autistic Behaviors

Despite the fact that spine density may increase or decrease as can be seen in Section “Many Upstream Regulators of mTORC1 are Expressed at the Dendrite or at the Post-Synaptic Density (PSD)”, there are consistent social behavior deficits reported in all rodent models of these upstream regulators of mTORC1 (Tables s 3, 4). Tests range from assays of social interaction/motivation such as the 3-chamber test or ultrasonic vocalization, social recognition tests such as social novelty, anxiety measured by the open field or the elevated plus maze testing to repetitive behaviors such as self-grooming and perservatory behaviors such as longer latencies to extinguish memories. Some tasks such as the Morris Water Maze also have a large cognitive component (Miyakawa et al., 2001; Holmes et al., 2002, 2003; Tsai et al., 2012).

### The Effects of Alterations of Positive Regulators of mTORC1 on Behaviors That Denote Autism

CDKL5 heterozygotes show reduced learning in the Barnes Maze and repetitive jumping behavior (Trazzi et al., 2018) while heterozygote reeler mice show decreased freezing to tone in renewal trials during fear conditioning (Iafrati et al., 2013). Similarly, the *Dip2A* KO shows lower social exploration time of a conspecific, increased self-grooming in both novel and home cage environments, decreased social novelty and increased marble burying indicative of higher anxiety as well as social recognition and interaction deficits (Ma et al., 2019).

Notably, rodent ASC models utilizing SHANK3 mutations have good face validity, reflecting both repetitive and altered social behavior phenotypes, including reliably increased self-grooming behavior, lower levels of social interaction and ultrasonic vocalization and increased anxiety in a domain-deletion independent manner (Wang et al., 2011; Jaramillo et al., 2016; Mei et al., 2016; Zhou et al., 2016). Most models also show decreased social recognition memory (Peça et al., 2011; Schmeisser et al., 2012; Kouser et al., 2013; Mei et al., 2016) with males scoring worse in this task than females (Wang et al., 2011). Furthermore, ankyrin-repeat and proline rich domain mutants show hypoactivity in the open field (Wang et al., 2011; Zhou et al., 2016). For the global regulators, though *MeCP2* overexpression in mice recapitulates Rett syndrome, it is harder to see autistic-type behaviors. However, a recent study using *MeCP2* overexpression in cynomolgus monkeys showed that these monkeys display repetitive circling in their cages and lower time with familiar or novel conspecifics, indicative of ASC (Liu et al., 2016). Similarly, *UBE3A* overexpression results in repetitive behaviors and social interaction deficits (Smith et al., 2011). In general, for positive regulators of mTORC1, tests of social interaction/recognition/motivation tend to report a deficit compared to tests of spatial memory where reversal of the memory is typically measured (Monteiro and Feng, 2017).

### The Effects of Alterations of Negative Regulators of mTORC1 on Behaviors That Denote Autism

*Pten* KO mice show lower interaction with conspecific mice or novel mice and hyperactivity in the open field (Kwon et al., 2006). Unusually, in GRIN2B^+^/C456Y mice that mimic a human ASC mutation, NMDAR-dependent long-term depression (LTD) is decreased and though there is normal social interaction, there is an anxiolytic effect as measured by the elevated plus maze; the molecular mechanisms underlying this phenotype remain obscure (Shin et al., 2020). A similar anxiolytic effect is seen in a comprehensive panel of behavioral tests of the haploinsufficient *SynGAP*^+/−^ mice which also show no deficit in social interaction but hyperactivity and cognitive deficits in working and reference memory (Ma et al., 2019) and in a dentate gyrus-specific context discrimination task (Clement et al., 2012). A similar deficit in spatial memory is seen in two *Shank2* PDZ-deletion models, combined with increased self-grooming behavior and anxiety along with decreased social interaction or novelty (Schmeisser et al., 2012; Won et al., 2012). Impaired social interaction and novel object recognition (NOR) along with hyperactivity is also seen in *Fmr1* KO mice (Qin et al., 2011; Bhattacharya et al., 2012). For negative regulators of mTORC1, it is notable that spatial, hippocampal-based tasks show deficit and hyperactivity is seen in several models.

## Molecular Mechanisms That Link Dysfunctions in Neuromorphology to Behavior

Despite mTORC1 under and over-activation in rodent models of autism due to mutations or loss of upstream positive and negative regulators, it is notable that these models show autistic-type behaviors. Therefore, it is of interest to understand if common molecular pathways downstream of mTORC1 may underlie autistic behaviors. One of the most prevalent ideas is alteration in the excitation-inhibition (E/I) ratio (Rubenstein and Merzenich, 2003) because of functionally altered excitatory (Gibson et al., 2008) and inhibitory synapses (Wöhr et al., 2015; Vogt et al., 2018), or in some cases alteration of both. Though somewhat simplistic, a number of reviews suggest this as an useful framework to understand neurodevelopmental disorders (Howell and Smith, 2019; Sohal and Rubenstein, 2019). One caveat to keep in mind is that alteration of E/I ratio could be a mechanism for homeostatic synaptic plasticity that arises because of the primary deficit caused by mutation in a gene that is a risk for ASC (Nelson and Valakh, 2015 and references therein).

Though E/I ratio can be altered by several mechanisms, reduction of GABAergic afferents has been shown in several models, including those with over-activation and under-activation of mTORC1. For example, a meta-analyses of different mouse ASC models including MeCP2 (a positive regulator of mTORC1) and FMRP (a negative regulator of mTORC1) reduction reveals a reduction of parvalbumin-positive (PV) GABAergic interneurons such as chandelier and basket calls in the CA1 hippocampus or somatosensory cortex suggesting increased excitability (Gogolla et al., 2009). Supporting the critical role of inhibitory afferents, a knockout of PV inhibitory interneurons results in mice whose behavioral anomalies recapitulate that seen in ASC, i.e., atypical social interactions and repetitive behavior (Wöhr et al., 2015; Filice et al., 2020). In four models of ASC including *Fmr1*^−/y^ and *Tsc2*^+/–^, both of which are negative regulators of mTORC1, there is greatly reduced inhibition and a small reduction in excitation measured in the feedforward circuit in L2/L3 pyramidal neurons of the somatosensory cortex, suggesting that E/I ratio is altered (Antoine et al., 2019) primarily due to decreases in inhibitory feedforward mechanisms onto the postsynaptic neuron. Also, hyperexcitability in the hippocampus of *Tsc1* KO is due to decreases in inhibitory afferents onto *Tsc1* KO neurons and not due to increases in intrinsic excitability, synaptic glutamatergic afferents, or regulation of Arc-dependent synaptic homeostasis (Zhao and Yoshii, 2019). However, the importance of an optimal level of inhibitory transmission is shown by a recent study whereby increasing hippocampal astrogenesis that in turn increases GABAergic transmission induces ASC-like behavior in mice such as lack of social preference in the 3-chambered test and an increase in repetitive grooming (Chen et al., 2021a). Contrary to the decreased inhibition seen in the above models, the granule cells in the dentate gyrus of the *Pten* KO show increased density of synapses, greater spike rates and are more sensitive to depolarizing input, demonstrating hyperexcitability (Williams et al., 2015). In addition, loss of PTEN causes increases in basal dendrites that are contacted by other mossy fiber axons leading to recurrent circuits (Pun et al., 2012). Though many models of ASC that are mutated in genes that are negative regulators of mTORC1 appear to show altered E/I ratio due to decreased inhibition, the *Pten* example indicates that this is not an universal mechanism.

### Upstream Regulators of mTORC1 Regulate Molecules at the PSD to Effect Changes in Synaptic Physiology

One of the reasons for E/I imbalance could be altered spine density/morphology or synaptic proteins, such as glutamate receptors at the PSD that are often altered in rodent models of autism that have alterations of the upstream regulator proteins of mTORC1.

*For positive upstream regulators of mTORC1*: Hyperexcitability and a higher E/I ratio in the CA1 is due to higher localization of GluN2B and SAP102 in *CdKl5*^−/y^ mice, as revealed by subcellular fractionation of the hippocampal PSD (Okuda et al., 2017). In *Shank3* KO mouse striatum, the disrupted interaction between Homer1b/c-mGluR5 also results in increased mGluR5 at the PSD. Antagonists of the mGluR5 receptor such as MPEP could reverse the increased self-grooming in these mice, suggesting that receptor density at the PSD is critical for behavior (Wang et al., 2016). In mice carrying SHANK3 deletion of different domains, several receptors such as NR1, NR2A, GluR2, and GluA2 are decreased (Monteiro and Feng, 2017 and references therein). Conversely, the decreased mEPSC amplitude in the *Dip2a* KO is due to a smaller number of NMDA subunits at the PSD (Ma et al., 2019). In MeCP2 overexpressing mice, despite an increase in both mGluR and NMDA receptors at the synapse in the somatosensory cortex (Chahrour et al., 2008; Jiang et al., 2013), there is a reduction in the EPSCs because NMDA/AMPA currents correlate to reduced GluN1 expression; this is not due to reduction in GABAergic interneurons (Sceniak et al., 2016) in the prefrontal cortex. Rather, this suggests, as borne out by brain activity *c-Fos* mapping, that reduced functional excitation rather than reduced inhibition drives the decrease in E/I ratio in these animals since this is reversed by ketamine, showing the importance of post-synaptic glutamate receptors (Kron et al., 2012). This could demonstrate that alteration in E/I ratio is driven by different mechanisms in different brain regions since reduction in the MeCP2 overexpressing somatosensory cortex is due to a reduction in GABAergic parvalbumin neurons in that region (Gogolla et al., 2009) but this is not true in the prefrontal cortex (Sceniak et al., 2016).

*For negative upstream regulators of mTORC1*: In *Shank2* PDZ-deletion mice, the predominant pathway driving the atypical (for a negative regulator) decrease in spine density and social interaction deficit appears to be NMDA hypofunction since cycloserine, a NMDA agonist, can restore functionality (Won et al., 2012). However, AMPA receptors are also important; for example, SynGAP dispersal from the synapse, results in loss of Ras-ERK inhibition and leads to subsequent mTOR-mediated increased AMPA receptor insertion at the PSD, a prerequisite for LTP. Therefore, in SynGAP-deficient mice, though the proportion of mature spines and basal synaptic transmission are enhanced, no further enhancement is possible since increased AMPA receptor insertion in the basal state occludes LTP (Gamache et al., 2020). A third negative regulator is *Tsc1/2*; a pan-neuronal KO of *Tsc1* shows reduced frequency of GABAergic mIPSC, resulting in increased excitability in the cortex and an increased E/I ratio; however, a PV-specific *Tsc1* KO does not show altered E/I balance, suggesting that the local hyperexcitability is not due to loss of TSC signaling in PV interneurons (Zhao and Yoshii, 2019). Similarly in the hippocampus, though homeostatic plasticity *via* decreased synaptic AMPA receptors is seen, in experiments where homeostatic plasticity is not initiated, the increased E/I ratio is primarily due to impaired mGluR5-LTD at hippocampal synapses that may be because of reduced Arc and other proteins in the *Tsc1* KO that are required to stabilize LTD (Bateup et al., 2013). In support of this idea, the *Tsc1* mutant phenotype of reduced LTD and context discrimination can be rescued by an Fmr1^−/y^ mutant that shows the opposite, i.e., exaggerated mGluR5-LTD, suggesting that levels of PSD proteins maintained by these mTORC1 regulators underlie the deficit despite the fact that loss of Tsc1 and FRMP proteins both lead to overactivation of mTORC1 (Auerbach et al., 2011). In the amygdala of the *Fmr1* KO mouse, principal cells show intrinsic hyperexcitability and increased synaptic plasticity (Svalina et al., 2021), suggesting that the enhancement of PSD-95 and *Arc* levels seen in the PSD density of spines of hippocampal neurons (Yan et al., 2018) may also be true of other limbic regions to result in greater excitability.

### Upstream Regulators of mTORC1 Regulate Spine Morphology and Density *via* Pruning and/or Spinogenesis

Though the relationship between spine density and synaptic physiology appears to be complex, looking at spine morphology paints a clearer picture (Tables s 3 and 4), with an increase in immature spines in many models. A number of knockout rodent models of negative regulators such as FMRP, GRIN2B, and PTEN show an increase in the proportion of thin or filopodial spines vs. mushroom spines. Similarly, in the *Dip2a* KO, there is an increase in spine density but a decrease in mushroom shaped spines and a flattening of the PSD with a decrease of NMDA receptors in a smaller spine volume, possibly leading to a lower E/I ratio (Ma et al., 2019).

Some of this could also be due to spine instability. This is seen in *Fmr1* KO mice where there is a developmental delay in the transition from immature to mature spines in Layer2/3 pyramidal neurons of the cortex; thin spines are unusually unstable. Normally, these spines search for presynaptic partners in a lengthening process facilitated by mGluR5; however, mGluR5 agonists are unable to lengthen spines and achieve this in *Fmr1* KO mice (Ginsberg et al., 2012) suggesting that stabilization *via* contact with pre-synaptic partners is deficient when FMRP is absent. Interestingly, spines are stabilized in an ERK-dependent manner solely in clusters whose numbers are increased in mice overexpressing MeCP2 resulting in a higher proportion of mushroom shaped spines; isolated spines are formed and stabilized at the same rate as the wildtype mouse. Such stabilization of spines at a young age is correlated with increased stability on the rotarod, a marker of perseverative behavior in these mice (Ash et al., 2021a, b) though it is unclear if this is cause of effect. For example, repetitive task learning that is cortex-dependent induced spines preferentially in clusters; these clustered spines showed greater persistence past learning than non-clustered spines (Fu et al., 2012) and hence the functional interpretation of spine clustering in ASC models is difficult to ascertain.

Spine morphology also has functional consequences; shRNA targeting *Pten* results in increased spine density in the basolateral amygdala (BLA) of the mouse with an increased mEPSC amplitude and frequency in the BLA. This is most likely due to a shift in spine morphology with an increased proportion of mushroom spines but decreased filopodia (Haws et al., 2014). Consistent with this finding, though granule cells of neonatal *Pten* KO mice show hypertrophy and therefore might be expected to be less excitable, increased density of mushroom shaped spines concomitant with increased dendrites and protrusions drives the hyperexcitability observed in this ASC model (Williams et al., 2015). This increase in spines is larger when *Pten* KO granule cells are sparsely surrounded by wildtype cells when compared to when they are densely surrounded by other *Pten* KO cells (Skelton et al., 2019) though the mechanism underlying this and its significance is unknown. It could be that secondary effects on network activity amongst surrounding cells and subsequent tertiary feedback effects by these neighboring cells on the *Pten* KO neuron drive the ASC phenotype (LaSarge and Danzer, 2014).

#### Upstream Regulators of mTORC1 Regulate Pruning by Several Pathways

Though Tang et al. elegantly showed that pruning deficits are critical for the increase in spine density in the *Tsc2*^+/−^ mice (Tang et al., 2014), other studies have also shown pruning deficits. In some cases, such as MeCP2 overexpression, 2-photon imaging of L5 apical dendrites emanating from cortical pyramidal neurons reveals a slowing down of pruning and increased spinogenesis with unstable spines as development proceeds, most likely due to mTORC1 overactivation (Jiang et al., 2013). Microglia-activated pruning is lower in the CA1 hippocampal neuron in a mouse FXS model (Jawaid et al., 2018). Delivery of shRNA to Raptor into the CA1 of FXS mice decreases the PSD-95 and ARC proteins that are elevated in these mice and decreases the spine density and reduces cognitive deficits. Importantly, shRNA to Raptor in *Fmr1* KO mice also decreases filopodial spines and increases the proportion of mushroom shaped spines (Yan et al., 2018). In *Fmr1* KO neurons, higher levels of eIF1α sequester the E3 ubiquitin ligase, murine double minute2 (mdm2) which targets PSD-95 for degradation. Hence, in response to neuronal activity in these *Fmr1* KO neurons, myocyte enhancer factor (MEF)-induced degradation of PSD-95 degradation and synapse elimination/pruning does not occur leading to higher spine densities (Pfeiffer et al., 2010). Therefore, in the *Fmr1* KO mouse, overactivation of mTORC1 directly results in a decrease in pruning and higher spine densities.

The converse—an increase in pruning leading to a decrease in spines also exists. For example, in an inducible UBE3A overexpression model of autism, an increase in pruning over spine growth results in a decrease in spines. UBE3A overexpression results in increased targeting and degradation of a caspase inhibitor, XIAP, which leads to increased caspase levels with concomitant increases in tubulin cleavage and spine retraction (Khatri et al., 2018). Conversely, expression of a dominant negative caspase could decrease tubulin cleavage and allow for more mature spines and increased spine density (Khatri et al., 2018).

#### Upstream Regulators of mTORC1 Regulate Spinogenesis by Several Pathways

Apart from pruning, a second pathway to alter spine density is spinogenesis, driven by several Rho/ROCK pathways that activate LIMK; these include the Rac/p21-activated kinase (PAK) pathways that can activate cortactin and/or LIM kinase (LIMK; Costa et al., 2020). For example, in the *Dip2A* KO which shows increased spine density but a flattened PSD, there is a reduction of acetylation of cortactin, a protein activated by PAK that increases spine stability (Schnoor et al., 2018); DIP2A mutations implicated in ASC are in regions of the protein that contact cortactin. Administration of an acetylated cortactin mimic into the cerebral cortex rescues not only the flattened PSD and decreases spine density but also decreases the self-grooming and marble burying behavioral phenotype (Ma et al., 2019). Similarly, a knockdown of all Shank isoforms using miRNA in rat hippocampal neurons resulted in a decrease in spine density (specifically of mushroom shaped spines) and cortactin levels, that could not be rescued by a SHANK2 isoform deficient in cortactin binding, suggesting that SHANK isoforms recruit and stabilize cortactin in the PSD (MacGillavry et al., 2016) to grow mushroom-shaped spines. Indeed, *Shank3* KO show lower levels of Rac1 and PAK proteins and an increase in actin depolymerization and behavioral ASC-like phenotypes in this mouse could be rescued by increasing Rac1, suggesting that actin dynamics and spine density were critical in driving behavior (Duffney et al., 2015). Interestingly, S6K activation activates Rac/PAK in ovarian cancer cells (Ip et al., 2011) while loss of mTORC1 leads to constitutive activation of ROCK in the epidermis (Asrani et al., 2017), suggesting that these pathways may also be present in the CNS, to regulate spinogenesis.

Amongst negative regulators of mTORC1 such as FMRP, a decrease in phosphorylated active SLINGSHOT protein, an inhibitor of LIMK combined with an increased phospho-LIMK due to increased Rac/PAK levels, leads to a decreased level of active cofilin and subsequent actin depolymerization (Pyronneau et al., 2017). This could be the mechanism that underlies the increase in filamentous spines in this model. Sequestration of proteins in the spinogenesis pathway is also a mechanism for regulation by mTORC1 regulators. For example, the cytoplasmic FMRP interacting protein (CYFIP1) sequesters and inhibits eIF4E to repress translation along with FMRP but this protein by itself is also part of the WAVE-Rac complex that increases spine protrusion. In the absence of FMRP in hippocampal neurons, the balance shifts towards spine protrusion and away from translation repression to increase spine growth, a phenomenon that can be reversed by the administration of 4EGI-1, which creates more free eIF4E that can bind CYFIP1 away from the WAVE complex (Santini et al., 2017). A caveat to these studies is that direct inhibition of spinogenesis pathways may be more important in some cases than regulation *via* mTORC1. NF-1, a negative regulator of mTORC1, leads to an overall decreased spine density when deleted in neurons, possibly due to inhibition of the ERK pathway which is required for spine stabilization (Ash et al., 2021a), suggesting that ERK-driven spinogenesis may supercede mTORC1-driven pruning. Indeed, as can be seen many of the upstream regulators of mTORC1 employ several mTORC1-independent means to regulate spine density.

### Upstream Regulators of mTORC1 May Preferentially Regulate Corticostriatal Circuitry

Corticostriatal circuitry which underlies motivated sensorimotor by interacting with the limbic system is the focus of several neurodevelopmental disorders, including autism (Shepherd, 2013), with several alterations to this circuit. In ASC individuals, intrinsic functional connectivity studies done using fMRI imaging shows that those with highly repetitive behaviors show reduced frontoparietal and motor connectivity with the striatum but increased connectivity with the limbic system compared to those with low repetitive behaviors, underscoring the importance of the corticostriatal circuit (Abbott et al., 2018). These differences in neuroanatomy particularly for the striatum are underscored by a longitudinal structural imaging study which revealed a faster growth rate of the caudate nucleus during late childhood to early puberty in ASC individuals that was strongly correlated with repetitive behaviors seen in these individuals that developed during the preschool period (Langen et al., 2014). These alterations in mice are linked to spines; conditional dorsal pallium-specific KOs of *Met*, a tyrosine kinase that regulates spinogenesis, demonstrates local hyperconnectivity in this circuit with stronger afferents from corticostriatal neurons in layer 2/3 to layer 5 projection neurons in the anterior frontal cortex in heterozygotes and KOs compared to WT controls (Qiu et al., 2011).

Developmental studies of *Shank3*, a positive regulator of mTORC1, show that the striatal excitability is higher due to increased cortical glutamatergic efferents to the striatum (Peixoto et al., 2016) at early postnatal stages but there is reduced corticostriatal connectivity and EPSCs as adults (Mei et al., 2016; Wang et al., 2016). In addition, disruption of the dopaminergic receptor D1R-SYNGAP complex in the prefrontal cortex during development results in decreased migration of GABAergic interneurons with concomitant increased cortical excitability. This cortical excitability that develops as a result of disruption of the function of SYNGAP and D1R is instrumental in the social deficits seen in this model during adulthood (Lai et al., 2021). In *Tsc2*^+/−^ mice, resting state fMRI reveals functional hyperconnectivity within the corticostriatal circuit due to increased synaptic coupling possibly as a result of increased spine density; this was strongly correlated to repetitive behaviors. All parameters, i.e., connectivity, spine density, synaptic coupling, resting state default mode network signatures, and social behaviors are rescued to normal levels with rapamycin, suggesting that mTORC1 overactivation in this circuit is crucial to spine dynamics that underlie social behaviors. This hyperconnectivity signature is valid cross-species—children with ASC showed similar fronto-insular-striatal hyperconnectivity and mTORC1 interacting genes are enriched in the ASC cortical transcriptome, suggesting that this *Tsc*-mTORC1 pathway is a critical driver of functional hyperconnectivity (Pagani et al., 2021). Cross-species connectivity is also shown with mutations of NF1, a negative regulator of mTOR similar to *Tsc1/2*, though *Nf1* KO mice show decreased spine density. Children with NF1 mutations with ASC show reduced functional connectivity between the striatum and the frontoparietal network and increased striatal functional connectivity with the limbic system. Similar to the human scenario, Nf1^+/−^ mice also show disrupted corticostriatal connectivity, as revealed by resting state imaging (Shofty et al., 2019). This shows that both positive, e.g., SHANK and negative regulators. e.g., TSC of mTORC1 typically show spine density alterations in the corticostriatal circuit that are commensurate with local connectivity signatures and may underlie the repetitive behaviors seen in both humans and rodent models.

## Conclusion/Future Directions

Despite a number of studies that show both spine dynamics and social behavior alterations in ASC rodent models, a number of questions persist. Many models are homozygous deletion models in order to obtain more robust effects whereas they tend to be haploinsufficient conditions in the human (Monteiro and Feng, 2017) and data from ASC rodent models that identify molecular processes that are dysregulated may not translate to the human. For example, though reduction in GABAergic transmission has been identified in FXS (Section “Upstream Regulators of mTORC1 Regulate Molecules at the PSD to Effect Changes in Synaptic Physiology”), use of a selective GABA-B receptor agonist arbaclofen showed limited improvements in social behaviors only in young but not older children and adults in a Phase 3 clinical trial (Berry-Kravis et al., 2017). Similarly, mTOR inhibition in TSC patients has shown improvements for epileptic symptoms (Mizuguchi et al., 2019) but not for ASD-associated behaviors (Overwater et al., 2019), suggesting that treatment must be given in the critical period or that mTOR signaling is less important in ASC symptoms in humans.

Furthermore, though several of the upstream regulators such as SynGAP and SHANK have isoforms, the differential contribution of isoforms is not clear and deserves more attention. For example, SynGAPα1 is the only isoform capable of being anchored at the PSD due to the presence of its unique C-terminal PDZ binding domain and is sufficient for the enlargement of spine heads by AMPA receptor insertion upon chemically induced LTP as well as increase in PSD-95-positive puncta. SynGAPβ isoform rescues the reduction in dendritic arborization seen in cultured hippocampal neurons when SynGAP levels are reduced (Araki et al., 2020). Compensation by isoforms also confounds interpretation of the results, particularly in the case of conserved SHANK proteins.

In addition to isoform-specific functions of mTORC1 upstream regulators, the role of these genes has typically been studied in two major brain regions, i.e., the hippocampus and cortex and region-specific differences exist (Figure 4). For example, in *Shank3*^−/−^ mice, mGluR5 is increased in the striatum but not in the cortex (Wang et al., 2016), possibly pointing to the importance of the corticostriatal synapse in modulation of social behaviors that have reward potential. *Shank3* mice deleted for the ankyrin repeat domain show reduced excitation but increased frequency of sIPSCs at Schaffer collateral synapses in pyramidal neurons in the hippocampus. However, prelimbic layer 2/3 pyramidal neurons in the medial prefrontal cortex show decreased frequency of sIPSCs, though the basis of this difference is not known (Lee et al., 2015). In *Shank2* PDZ-deletion mutant mice, NMDA hypofunction also leads to lower LTD and lower LTP at Schaffer collateral synapses but not in the prefrontal cortex (Won et al., 2012), suggesting that microcircuitry in the hippocampus and cortex may lead to different E/I ratios. There are also cell-specific differences in regulation. For example, FMRP transcripts are greatly reduced in cerebellar Purkinje cells in the *Tsc1* mutant (Dalal et al., 2021) and theoretically could lead to elevated levels of FMRP target genes such as mTOR (Casingal et al., 2020), leading to an overactivation of mTORC1. However, although FMRP functions as a general translational repressor, it may also regulate ribosome binding to specific transcripts; indeed a recent study in cortical neurons saw destabilization of specific but not all transcripts (Shu et al., 2020) despite FMRP loss. However, in hippocampal slices derived from Tsc2^+/−^ mice, there was increased expression of FMRP targets (Hien et al., 2020), most likely due to differences in *Tsc-Fmr1* regulation in the cerebellum vs. the hippocampus. Apart from the hippocampus, cortex and cerebellum, many social behaviors are regulated by nuclei in the social behavior network which includes hypothalamic nuclei (Newman, 1999; Greenberg and Trainor, 2016) in a sexually dimorphic manner and spine dynamics in these regions in these ASC models is under-explored. Given the difference in incidence of ASC symptoms between the sexes with males showing greater incidence than females (Zhang et al., 2020), this would be timely to investigate.

Though the picture for changes in spine dynamics upon deletion or duplication of these genes is complex, it can be simplified when seen through the lens of regulation of mTORC1 as part of a larger grouping (Tables s 3 and 4); this is especially true of positive regulators of mTORC1 (Table 3; Figures 4C,F). For some genes such as FMRP, it is clear that both mTORC1-dependent and mTORC1-independent means of regulating spine density and synaptic physiology exist with the contribution of each not clearly understood though the weight of studies suggest an overall increase in spine density (Figure 3). In addition, spine density and in particular spine morphology studies tend to be time consuming and analyses is frequently done manually. In the coming years, it would be advantageous to investigate spine dynamics using 2-photon imaging as well as artificial intelligence algorithms (Mancuso et al., 2013) for additional information on spine motility and stability, including spine clustering, in several of these rodent ASC models to probe the relationship between spine morphology, stability, neurocircuitry, and behavior.

## Author Contributions

NV framed and analyzed most of the scientific content in the manuscript. SC did the analyses of individual mTORC1 regulators including the summary of the spine density and morphology in tabular form. Both SC and NV discussed and wrote the manuscript. All authors contributed to the article and approved the submitted version.

## Conflict of Interest

The authors declare that the research was conducted in the absence of any commercial or financial relationships that could be construed as a potential conflict of interest.

## Publisher’s Note

All claims expressed in this article are solely those of the authors and do not necessarily represent those of their affiliated organizations, or those of the publisher, the editors and the reviewers. Any product that may be evaluated in this article, or claim that may be made by its manufacturer, is not guaranteed or endorsed by the publisher.
